# A sandwich-type electrochemical immunosensor based on the biotin- streptavidin-biotin structure for detection of human immunoglobulin G

**DOI:** 10.1038/srep22694

**Published:** 2016-03-07

**Authors:** Yueyun Li, Yihe Zhang, Liping Jiang, Paul K. Chu, Yunhui Dong, Qin Wei

**Affiliations:** 1 Beijing Key Laboratory of Materials Utilization of Nonmetallic Minerals and Solid Wastes, National Laboratory of Mineral Materials, School of Materials Science and Technology, China University of Geosciences, Beijing, 100083, P. R. China; 2 School of Chemical Engineering, Shandong University of Technology, Zibo, 255049, P. R. China; 3 Department of Physics & Materials Science, City University of Hong Kong, Tat Chee Avenue, Kowloon, Hong Kong, China; 4 Key Laboratory of Chemical Sensing & Analysis in Universities of Shandong, School of Chemistry and Chemical Engineering, University of Jinan, Jinan, 250022, P.R. China

## Abstract

A sandwich-type immunosensor is designed and fabricated to detect the human immunoglobulin G (HIgG) using polyaniline and tin dioxide functionalized graphene (GS-SnO_2_-PAN) as the platform and biotin-functionalized amination magnetic nanoparticles composite (B-Fe_3_O_4_@APTES) as the label. GS-SnO_2_-PAN is used as the sensing agent to capture the primary anti-HIgG (Ab_1_) and SnO_2_ reduces the stack of GS. The B-Fe_3_O_4_@APTES with a large surface area and excellent biocompatibility captures second antibody (Ab_2_) efficiently based on the highly selective recognition of streptavidin to biotinylated antibody. The B-Fe_3_O_4_@APTES has better electro-catalytic activity in the reduction of hydrogen peroxide (H_2_O_2_) and the “biotin-streptavidin-biotin” (B-SA-B) strategy leads to signal amplification. Under optimal conditions, the immunosensor has a wide sensitivity range from 1 pg/L to 10 ng/L and low detection limit of 0.33 pg/L (S/N = 3) for HIgG. The immunosensor has high sensitivity, fast assay rate, as well as good reproducibility, specificity, and stability especially in the quantitative detection of biomolecules in serum samples.

Immunoglobulin (Ig) is an important component in the immune system and plays an important role in recognizing bacteria and viruses. There are five types of Igs in the human plasma and human immunoglobulin G (HIgG) is the most important one accounting for about 75% of the total Igs^1^,^2^. HIgG is effective in the treatment of humoral mediated neurological autoimmune diseases and has been reported to improve the outcome in some neuromyelitis optica patients^3^.

Electrochemical immunosensors based on the antigen-antibody specific reactions have recently attracted much interest and many immunosensors have been used for the detection of HIgG^4^,^5^. Compared to conventional immunoassays such as enzyme linked immunosorbent assays (ELISA)^6^, fluoroimunoassays^7^, and chemiluminescence immunoassays^8^, electrochemical immunosensors are excellent in the detection of disease-related proteins due to inherent advantages including simple pretreatment procedures, short analytical time, precise current measurements, and inexpensive instrumentation^9^^–^12. In order to improve the sensitivity and selectivity of electrochemical immunosensors, interface materials serving as good carriers or bridges for efficient immobilization of proteins are critical^13^. The physical and chemical properties of the interface play a crucial role in the assay performance^13^,^14^.

SnO_2_ nanoparticles are used widely in biosensors on account of the high electron mobility as well as good chemical and thermal stability^15^ and graphene sheets (GS) have excellent conductivity and large specific surface area. Yao *et al*. developed an *in situ* synthesis method to produce tin dioxide - graphene (GS-SnO_2_) nanocomposite as the anode in lithium-ion batteries^16^ and Lu *et al*. investigated the electrochemical behavior of GS-SnO_2_ composite films in surpercapacitors^17^. However, there have been few reports on the use of GS-SnO_2_ nanocomposite in electrochemical sensors. SnO_2_ nanoparticles could be distributed on GS followed by the *in situ* synthesis of polyaniline (PAN) on the GS-SnO_2_ composite to obtain polyaniline functionalization of tin dioxide/graphene (GS-SnO_2_-PAN). PAN improves electron transfer due to the good electrical conductivity. SnO_2_ and PAN adhere on the GS to reduce the GS stack and form a sandwich-like structure composite. The GS-SnO_2_-PAN can be used as the sensing agent for immobilization of primary antibody (Ab_1_) to improve the electrochemical performance of the modified electrode.

An amplification strategy has been proposed to improve the sensitivity of electrochemical immunosensors using the biotin labeled protein-streptavidin (SA) network complex. SA, a 66 kDa protein has been widely used in immunohistochemistry^18^ and immunoassay^19^ due to its high specificity and strong affinity for biotin^20^. In the immunoassay, the high affinity of SA and biotin^21^ benefits the capture of specific classes of proteins^22^.

In this work, an electrochemical immunosensor is described for quantitative detection of HIgG by using the biotin-functionalized amination magnetic nanoparticles composite (B-Fe_3_O_4_@APTES) and GS-SnO_2_-PAN for signal amplification. The B-Fe_3_O_4_@APTES is used as a label for Ab_2_ through catalyzing the electrochemical reaction of hydrogen peroxide (H_2_O_2_). The 3-aminopropyltriethoxysilane (APTES) functionalized Fe_3_O_4_ (Fe_3_O_4_@APTES) can bind biotin through the exposed active amino groups and carboxy group on biotin^23^. This “biotin-streptavidin-biotin” (B-SA-B) structure is utilized to combine Ab_2_ and the marker (B-Fe_3_O_4_@APTES). The cross-shaped SA has one free biotin-binding site available for a biotinylated antibody (B-Ab_2_) and the other three binding sites are conjugated with a 3 equimolar ratio of B-Fe_3_O_4_@APTES to achieve triple-amplification. Additionally, the B-Fe_3_O_4_@APTES can be connected repeatedly by SA connection SA/B-Fe_3_O_4_@APTES for further multiple amplification signals. The novel immunosensor shows high sensitivity, fast assay speed, wide linear detection range, and low detection limit has potential applications in quantitative detection of HIgG.

## Results and Discussion

### Characterization of GS-SnO_2_-PAN composites

**Figure 1:**
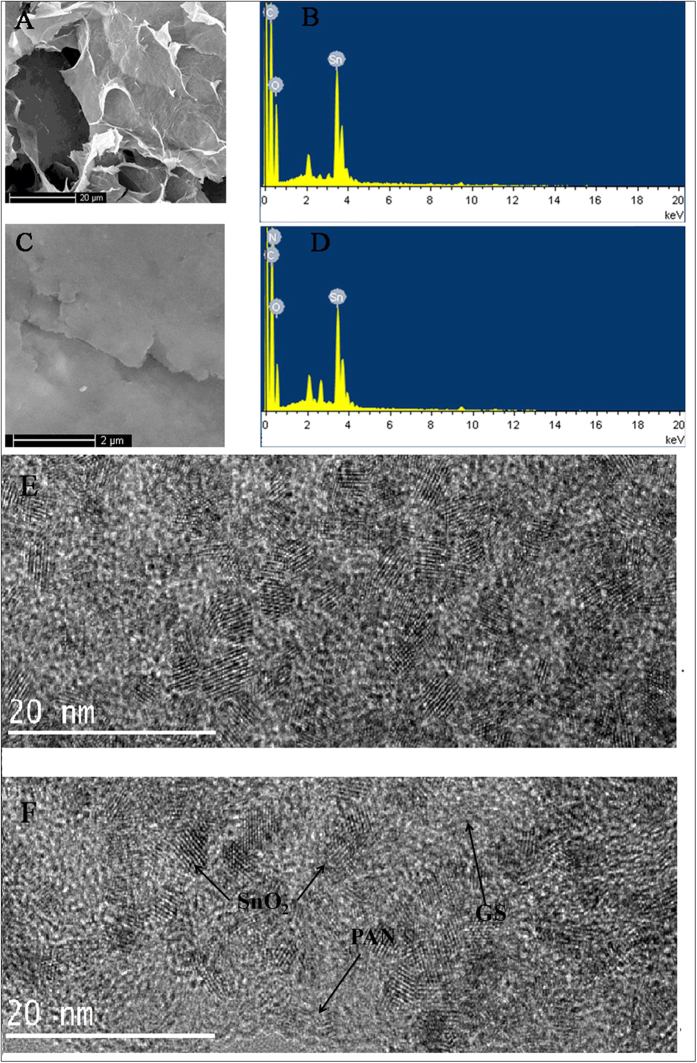
(**A**) SEM image of GS-SnO_2_; (**B**) EDS spectrum of GS-SnO_2_; (**C**) SEM image of GS-SnO_2_-PAN; (**D**) EDS spectrum of GS-SnO_2_-PAN; (**E**) HR-TEM image of GS-SnO_2_; (**F**) HR-TEM image of GS-SnO_2_-PAN.

**Figure 2:**
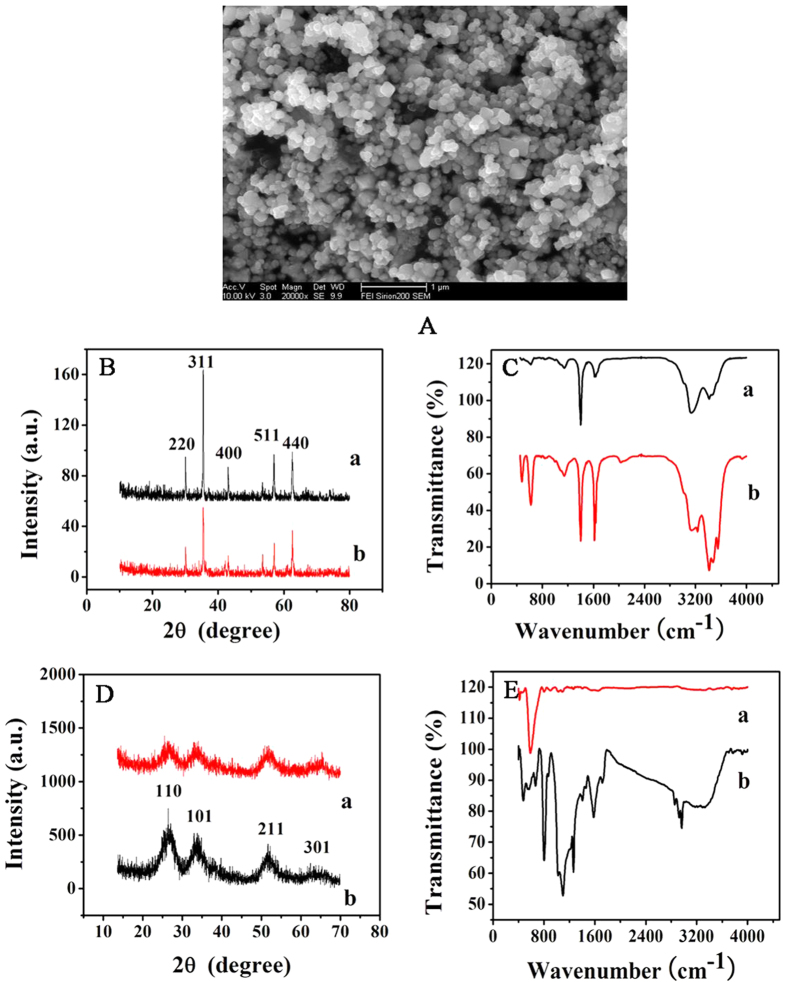
(**A**) SEM image of magnetite nanoparticles Fe_3_O_4_@APTES; (**B**) XRD spectra of (a) Fe_3_O_4_ and (b) Fe_3_O_4_@APTES; (**C**) FTIR spectra of (a) Fe_3_O_4_ and (b) Fe_3_O_4_@APTES; (**D**) XRD images of (a) GS-SnO_2_-PAN and (b) GS-SnO_2_; (**E**) FTIR spectra of (a) GS-SnO_2_-PAN and (b) GS-SnO_2_.

The morphology of GS-SnO_2_ and GS-SnO_2_-PAN is examined by SEM. As shown in Fig. 1A, irregular SnO_2_ grains are distributed on the GS which resembles a piece of wrinkled paper. The SnO_2_ nanoparticles with positive surface charges in a specific pH range can interact with GS by physical sorption, electrostatic binding, or charge transfer to produce a sandwich structure^24^. The EDS results reveal C, O, and Sn (Fig. 1B) and the presence of SnO_2_ can prevent stacking of GS layers and improve the dispersion. As shown in Fig. 1C, GS-SnO_2_-PAN has a smooth surface and the white SnO_2_ nanoparticles are covered by PAN. EDS conducted on GS-SnO_2_-PAN (Fig. 1D) confirms the presence of PAN showing an obvious N signal. The PAN increases the distance between layers of GS-SnO_2_ and Ab_1_ can better be immobilized on the GS-SnO_2_-PAN. The TEM image of GS-SnO_2_ (Fig. 1E) and GS-SnO_2_-PAN (Fig. 1F) characterized the dispersion of the SnO_2_, GS and PAN. As shown in Fig. 1E,F, it can be seen that SnO_2_ with diameters of approximately 4 nm evenly dispersed on the surface of GS. The lattice structure of PAN, GS, SnO_2_ is evidently. The XRD patterns of the GS-SnO_2_ nanocrystals and as-prepared GS-SnO_2_-PAN composites are shown in Fig. 2D. Figure 2D(a) shows the XRD pattern of the GS-SnO_2_ composite. The major diffraction peaks from bare SnO_2_ at 26.5°, 33.9°, 51.8°, and 65.8° can be indexed to (110), (101), (211), and (301) of the tetragonal SnO_2_ nanocrystals^25^,^26^. The broad diffraction patterns indicate small particle size of SnO_2_. The GS shows the (100) diffraction peak^27^ which coincides with the (110) diffraction peak of SnO_2_. There are no observable impurity peaks and no graphite peak at 26.6° is observed from GS-SnO_2_, suggesting that agglomeration of GS is inhibited by the SnO_2_ nanoparticles on the surface and good dispersion in the composites. Figure 2D(b) shows the XRD pattern of the GS-SnO_2_-PAN composite. The peak positions are consistent but the intensity of GS-SnO_2_-PAN is lower than that of GS-SnO_2_ T because of introduction of PAN to the surface. The GS-SnO_2_-PAN shows only one weak broad (002) peak in Fig. 2D(b) and it may be due to the corrugated pore structure of GS-SnO_2_-PAN^28^,^29^,^30^.

In order to evaluate the formation of GS-SnO_2_-PAN composites, FTIR is performed (Fig. 2E). With regard to PAN, the peaks at 1562 and 1485 cm^−1^ correspond to the C=C stretching modes in quinoid and benzenoid ring whereas those at 1297, 1130 and 799 cm^−1^ are attributed to C−N stretching, C=N stretching, and C−C stretching in the benzenoid ring, respectively^31^,^32^. As shown in Fig. 2E, the peaks from the GS-SnO_2_-PAN composite are characteristic ones for PAN and Sn−O stretching indicating successful synthesis of GS-SnO_2_-PAN.

### Characterization of Fe_3_O_4_@APTES composites

**Figure 3:**
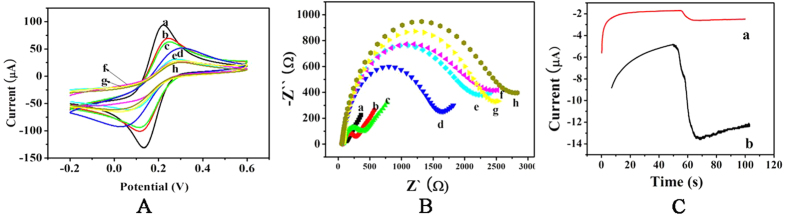
(**A**) CVs of the GCE (curve a), GS-SnO_2_-PAN/GCE (curve b), Ab_1_/GS-SnO_2_-PAN/GCE (curve c), BSA/Ab_1_/GS-SnO_2_-PAN/GCE (curve d), HIgG/BSA/Ab_1_/GS-SnO_2_-PAN/GCE (curve e), B-Ab_2_/HIgG/BSA/Ab_1_/GS-SnO_2_-PAN /GCE (curve f), SA/B-Ab_2_/HIgG/BSA/Ab_1_/GS-SnO_2_-PAN /GCE (curve g), Fe_3_O_4_@APTES-B/SA/B-Ab_2_/HIgG/BSA/Ab_1_/GS-SnO_2_-PAN/GCE (curve h); (**B**) Nyquist diagrams of electrochemical impedance spectra recorded from 0.1 to 105 Hz of bare GCE (curve a), GS-SnO_2_-PAN/GCE (curve b), Ab_1_/GS-SnO_2_-PAN/GCE (curve c), BSA/Ab_1_/GS-SnO_2_-PAN/GCE (curve d), HIgG/BSA/Ab_1_/GS-SnO_2_-PAN/GCE (curve e), B-Ab_2_/HIgG/BSA/Ab_1_/GS-SnO_2_-PAN/GCE (curve f), SA/B-Ab_2_/HIgG/BSA/Ab_1_/GS-SnO_2_-PAN/GCE (curve g), Fe_3_O_4_@APTES-B/SA/B-Ab_2_/HIgG/BSA/Ab_1_/GS-SnO_2_-PAN/GCE (curve h) modified electrode in PBS containing 2.5 mM K_3_Fe(CN)_6_; (**C**) Comparison between multiple amplification amperometric responses of (a) one-off modified and (b) multiple replication modified electrodes.

The morphology of the Fe_3_O_4_@APTES (Fig. 2A) is examined by scanning electron microscopy (SEM). As shown in Fig. 3, the Fe_3_O_4_@APTES particles were an approximate spherical shape whose average diameter is 100 nm. XRD is performed on the Fe_3_O_4_ and Fe_3_O_4_@APTES nanoparticles (Fig. 2B). The diffraction peaks are broadened due to the small crystallite size. The diffraction peaks in Fig. 2B (a) (2*θ* =  30.2°, 35.5°, 43.1°, 57.1° and 62.8°) correspond to (111), (220), (311), (400), (511), and (440) of Fe_3_O_4_^33^,^34^. The average crystallite size is 100 nm. The same characteristic peaks can be found from Fig. 2B(b) illustrating that the characteristic peaks do not change but only the peak intensity and width after coating with amino-silane indicating that the crystalline structure of the modified nanoparticles is not varied. In addition, the coating process does not cause growth to and affect the physical properties of the magnetite particles^35^. No impurities are detected.

The FTIR spectra acquired from Fe_3_O_4_ and Fe_3_O_4_@APTES are depicted in Fig. 2C. The peaks at 563 and 580 cm^−1^ represent characteristic absorption of Fe−O confirming the presence of magnetite nanoparticles^36^,^37^, but the Fe−O−Si bond cannot be observed from Fig. 2C(a). It appears at around 578 cm^−1^ and overlaps the Fe−O vibration of magnetite nanoparticles^38^,^39^. Adsorption of silane polymer onto the magnetite particles is confirmed by bands at 1111, 1047, and 1018 cm^−1^ which correspond to the Si−O−H and Si−O−Si groups. The bands at 895 and 794 cm^−1^ are due to stretching of Si−O−H and vibration of OH on the surface of magnetite. For the Fe_3_O_4_@APTES, Si–O–Si stretching is verified by the band at 1122 cm^−1 ^39. The two broad band at 3419 and 1653 cm^−1^ can be ascribed to the N–H stretching vibration and NH_2_ bending of the free NH_2_ group, respectively^40^,^41^. As shown in Fig. 2C(b), the broad stretching peak at 3430 cm^−1^ is the bending mode of free NH_2_ groups in APTES^42^. The peak at 1431 cm^−1^ is related to the methylene group and that at 1664 cm^−1^ indicates C=O stretching in the carboxyl group^43^. The results show that the Fe_3_O_4_ is functionalized with amino groups and consistent with previous reports^44^,^45^.

### Characterization of the immunosensor

To characterize the sandwich-type immunosensor, CV is conducted in 5 mM K_3_[Fe(CN)_6_] solution (Fig. 3A). The GS-SnO_2_-PAN (curve b) is modified on a bare GCE (curve a) and the redox peak decreases because of lower electrical conductivity. Then Ab_1_ (curve c), BSA (curve d), HIgG (curve e), and B-Ab_2_ (curve f) are modified layer-by-layer on the electrode and the redox peak current declines gradually. The results suggest that the non-conductive bioactive substances reduce the efficiency of electron transfer. Finally, the SA (curve g) and B-Fe_3_O_4_@APTES (curve h) are modified on the electrode surface and the peak current decreases again demonstrating successful capture of SA and B-Fe_3_O_4_@APTES and that the immunocomplex inhibits electron transfer.

The A.C. impedance method is adopted to characterize the sandwich-type electrochemical immunosensor (Fig. 3B). The Nyquist plots are acquired from 1 to 10^5^ Hz at 0.24 V in the solution containing 0.1 M KCl and 2.5 mM Fe(CN)_6_^3−^/Fe(CN)_6_^4−^. The high-frequency region of the impedance plot shows a semicircle related to the redox probe Fe(CN)_6_^3−^/Fe(CN)_6_^4−^ and the semicircle diameter is equal to the resistance. The Warburg line in the low-frequency region corresponds to the diffusion step of the overall process. The resistance can be estimated from the diameter of the semicircle part at higher frequencies in the Nyquist plot. The bare GCE (curve a) shows a small resistance. Gradual increase in the impedance of the electrode surface with addition of GS-SnO_2_-PAN (curve b), Ab_1_ (curve c), BSA (curve d), HIgG (curve e), B-Ab_2_ (curve f), SA (curve g), and B-Fe_3_O_4_@APTES (curve h) demonstrates that biologically active substances hinder electron transfer between the working electrode and electrolyte implying successful capture.

In order to demonstrate the multiple amplification effect of the B-SA-B system, the one-off modified immunosensor is further modified with SA and B-Fe_3_O_4_@APTES. The amperometric i-t curve (Fig. 3C) shows that the catalytic ability of the modified immunosensor (b) is 8 times than that of the one-off modified immunosensor (a).

### Optimization of experimental conditions

**Figure 4:**
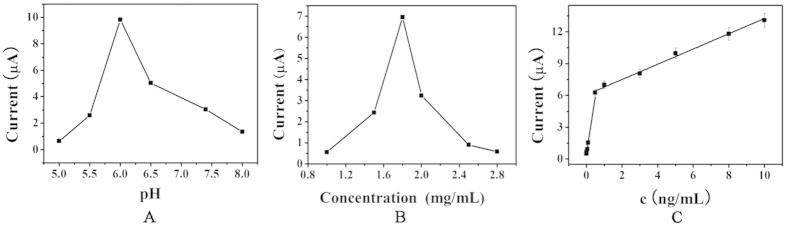
Effects of pH (**A**) of detection solution and concentration of Fe_3_O_4_@APTES (**B**) on the immunosensor. Error bar = 5%; (**C)** Calibration plot between the i-t current and the logarithm values of HIgG concentrations from 1 pg/mL to 0.5 ng/mL (0.001, 0.005, 0.01, 0.05, 0.1 and 0.5 ng/mL of HIgG, respectively) and from 0.5 ng/mL to 10 ng/mL (0.5, 1.0, 3.0, 5.0, 8.0, 10 ng/mL of HIgG, respectively).

To obtain optimal electrochemical signals, optimization of the experimental conditions including pH and Fe_3_O_4_@APTES concentration is necessary. The pH of the solution has a significant effect on the electrochemical behavior of the immunosensor because the activity of the antigen and antibody may be influenced by the highly acidic or alkaline surroundings^46^,^47^. In order to optimize the pH, a series of PBS with the pH from 5.0 to 8.0 is prepared. As shown in Fig. 4A, the current increases initially, reaches a maximum value at a pH of 6.0, and then decreases. Therefore, 6.0 is the optimal pH.

To accomplish sensitive detection of HIgG, the concentration of the Fe_3_O_4_@APTES is optimized. Different concentrations of Fe_3_O_4_@APTES (0.5, 0.75, 1.0, 1.5, 1.8, and 2.0 mg/mL) are used in the fabrication of the immunosensors. Figure 4B shows that the peak current increases from 1.0 to 1.8 mg/mL of Fe_3_O_4_@APTES. Because higher concentrations of Fe_3_O_4_@APTES affect the catalytic performance in the reduction of H_2_O_2_ and interface electron transfer resistance, the optimal concentration of Fe_3_O_4_@APTES is 1.8 mg/mL.

### Calibration curve

By adopting the optimal conditions, the sandwich-type immunosensor is utilized to determine different concentrations of HIgG in the PBS solutions based on the amperometric *i-t* current. Figure 4C shows the amperometric *i-t* current of different concentrations of HIgG in the PBS with a pH of 6.0. The current response versus concentration of HIgG shows good linearity in the range between 0.001 and 0.5 ng/mL as well as 0.5 ng/mL and 10 ng/mL with a detection limit of 0.33 pg/mL (S/N = 3). The regression equations of the calibration curves are Y_1_ = 0.4916 + 10.749×_1_ (R_1_ = 0.9777) and Y_2_  = 6.0646 + 0.7194×_2_ (R_2_ = 0.9901), respectively. The results demonstrate quantitative detection of HIgG.

### Comparison of different methods

Table S1 compares this method with others. The detection limit of this immunosensor is significantly lower than those of other methods. The lower detection limit is attributed to the triple amplification of SA-B-Fe_3_O_4_@APTES.

### Reproducibility, selectivity, and stability

**Figure 5:**
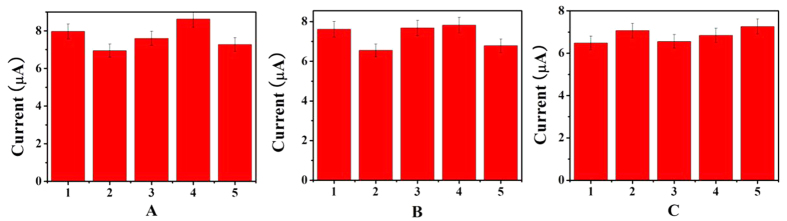
(**A**) Amperometric change response of the biosensor to different electrodes treated in same way to evaluate the reproducibility (RSD = 3.20%,); (**B**) Amperometric response of the immunosensor to 1.0 ng/mL HIgG (1), 1.0 ng/mL HIgG + 100 ng/mL AFP (2), 1.0 ng/mL HIgG + 100 ng/mL CEA (3), 1.0 ng/mL HIgG + 100 ng/mL PSA (4), 1.0 ng/mL HIgG + 100 ng/mL HIgE (5); (**C**) Stability study of the HIgG immunosensor.

To investigate the precision of the measurement, the electrochemical immunosensor is used to determine 1 ng/mL of HIgG (Fig. 5A). The relative standard deviation (RSD) is 3.20% indicating good precision and reproducibility. The influence of coexisting substances on the determination of HIgG is investigated by means of the amperometric i-t currents (Fig. 5B). The interfering substances include alpha fetal protein (AFP), carcinoembryonie antigen (CEA), prostate specific antigen (PSA), and humanimmunoglobulin E (HIgE). Compared to HIgG (1 ng/mL), the current change caused by introduction of the four proteins is less than 5% and so the selectivity is acceptable. The stability is also investigated by keeping the electrode at 4 °C when it is not in use. Figure 5C shows that 96.3% of the initial current response is retained after 2 weeks and 89.8% after 1 month indicating good stability in the buffer solution.

### Real sample analysis

In order to assess the clinical potential of the immunosensor, it is employed to detect the concentrations of HIgG in real serum sample according to the standard addition method. Table S2 shows the experimental results showing RSD between 1.4% and 2.4% and recovery in the range from 98.8% to 100.2%. The results demonstrate that the novel sandwich-type electrochemical immunosensor based on GS-SnO_2_-PAN as a platform and B-Fe_3_O_4_@APTES as the label for triple signal amplification for quantitative detection of HIgG has good reproducibility, selectivity, and stability and is clinically acceptable.

## Methods

### Reagents and Apparatus

The HIgG antibody (anti-HIgG, 12 mg/mL) and HIgG were bought from Dingguo Changsheng Biotechnology Company (Bei Jing, China) and bovine serum albumin (BSA, 96–99%) was obtained from Sigma (St. Louis, MO, USA). Streptavidin (SA) was purchased from Aladdin industrial corporation (China) and biotinamidohexanoic acid N-hydroxysuccinimide (B-NHS) ester and 3-aminopropyltriethoxysilane (APTES, 99%) were obtained from Sigma (St. Louis, MO, USA). Tin dioxide loaded graphene (SnO_2_-GS) (SnO_2_ content: 50 wt%, graphene sheet size: 500 nm~5 μm) was purchased from Xian Feng Nanomaterials Technology Company (Nanjing, China) and ferric oxide (Fe_3_O_4_) was purchased from Bo Di Chemical Industry Company (Tian Jin, China). The phosphate buffered solution (PBS, pH = 7.4) was prepared using 1/15 M KH_2_PO_4_ and 1/15 M Na_2_HPO_4_. The HIgG was stored at 4 °C and the standard solution was prepared daily with PBS. 5 mM K_3_[Fe(CN)_6_] was used as electrolyte for all electrochemistry measurements. All other chemicals were analytical reagents grade and used without further purification.

The electrochemical measurements were carried out on a CHI 760D electrochemical workstation (Shanghai CH Instruments Co., China) and cyclic voltammetry (CV) experiments were recorded in 5 mM K_3_[Fe(CN)_6_] by scanning the potential from −0.6 V to 0.2 V. In the impedance measurements, a frequency range of 100 kHz to 0.01 Hz and AC applied potential difference amplitude of 5 mV were used. Scanning electron microscopy (SEM) was conducted on the Quanta FEG250 field emission environmental SEM (FEI, United States) at 4 kV. The FTIR spectra were acquired on the FT-IR-410 infrared spectrometer (JASCO, Japan). X-ray diffraction (XRD) was performed on the D/Max 2500V/PC diffractometer (Rigaku Corporation, Japan) with Cu K_α_ irradiation (λ = 0.154 nm) at a scanning rate of 0.0202 θ/s and 2θ between 5°and 80°.

### Preparation of GS-SnO_2_-PAN composites

The black precipitate, GS-SnO_2_, was dispersed in dimethyl formamide (DMF, 10 mL) and aniline (0.05 mL) and hydrochloric acid (1 mL) were added to the suspension and stirred for 1 h. Ammonium persulfate (0.114 g) was added and the solution was ultra-sonicated for 12 h. After the reaction, GS-SnO_2_-PAN was obtained by washing with deionized water and ethanol and drying at 60 °C in a vacuum for 24 h.

### Preparation of biotinylated antibody (B-Ab_2_)

The biotinylated antibody was prepared according to the procedures described by Zhao *et al*.^48^ with some modifications as follows. The antibody solution (10 μg/mL) was produced with 1/15 mol/L PBS (pH = 7.4) and then mixed with 50 μL of 1.0 mg/mL B-NHS in dimethylsulfoxide (DMSO). The mixture was stirred for 4 h at room temperature and kept overnight at 4 °C. After dialyzing against PBS for 3 days, it was stored at 4 °C before use.

### Preparation of biotinylated magnetic nanoparticles (B-Fe_3_O_4_@APTES)

The Fe_3_O_4_ nanoparticles (0.5000 g) were dispersed in ethanol/water (volume ratio 1:1, 50 mL) ultrasonically for 30 min and then APTES (6 mL, 99%,) was added to the mixture under mechanical stirring at 40 °C for 8 h. The suspended magnetic nanoparticles were separated magnetically and the product (Fe_3_O_4_@APTES) was rinsed with ethanol 3 times to remove unreacted APTES molecules followed by drying at room temperature in vacuum. The Fe_3_O_4_@APTES (10.00 g) was dispersed in PBS (30 mL, pH = 7.4) and B-NHS (1 mg) dissolved in DMF (1 mL) was added under agitation. The B-Fe_3_O_4_@APTES solution was obtained after stirring at 37 °C for 12 h.

### Fabrication of the immunosensor

**Figure 6:**
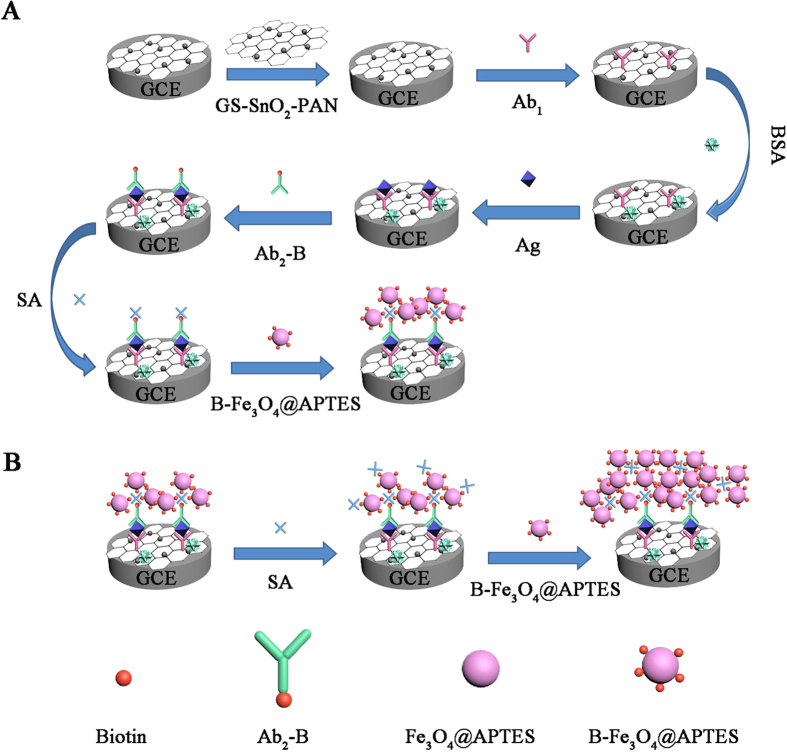
(**A**) Preparation procedures for the sandwich-type immunosensor and (**B**) Multiple amplification strategy by repeating the B−SA−B process.

Figure 6A shows the process of the fabrication of the sandwich-type electrochemical immunosensor and Fig. 6B illustrates the process for multiple-amplification by repeating the B−SA−B process. Generally, the GCE was polished by alumina powders with particle size of 1.0, 0.3, and 0.05 μm sequentially, followed by ultrasonic treatment in ethanol and rinsing with ultrapure water. The GS-SnO_2_-PAN (1 mg/mL, 6 μL) composite was put onto the pretreated electrode surface and dried. The GS-SnO_2_-PAN/GCE was incubated with Ab_1_ (10 μg/mL, 6 μL) and dried at 4 °C. After washing, 3 μL of 1 wt% BSA was modified onto the electrode and incubated for 0.5 h to eliminate nonspecific binding sites. The electrode was washed and incubated with different concentrations of HIgG for 1 h at room temperature and the electrode was washed extensively to remove unbounded HIgG molecules. Subsequently, the prepared B-Ab_2_ solution (10 μg/mL, 6 μL) was added to the surface of the electrode and SA (0.1 ng/mL, 6 μL) was modified on the surface of the electrode. Finally, the conjugated biotin (B-Fe_3_O_4_@APTES, 2 mg/mL, 6 μL) was added to the surface of the electrode to produce the electrochemical immunosensor.

### Detection of HIgG

A conventional three-electrode system was adopted in the electrochemical measurements. The GCE 4 mm in diameter was the working electrode, the saturated calomel electrode (SCE) was the reference electrode, and the platinum wire electrode served as the counter electrode. The PBS with a pH of 6.0 was used in the electrochemical measurements. CV was performed using a conventional electrochemical cell while scanning the potential from −0.2 to 0.6 V. The amperometric *i–t* curve was used to monitor the electrochemical signal in PBS by scanning the potential at −0.4 V and H_2_O_2_ (5 M, 10 μL) was added to PBS (10 mL) after the current stabilized under stirring.

## Additional Information

**How to cite this article**: Li, Y. *et al*. A sandwich-type electrochemical immunosensor based on the biotin- streptavidin-biotin structure for detection of human immunoglobulin G. *Sci. Rep.* **6**, 22694; doi: 10.1038/srep22694 (2016).

## Supplementary Material

Supplementary Information
