# Supplementary material for: A sandwich-type electrochemical immunosensor based on the biotin- streptavidin-biotin structure for detection of human immunoglobulin G
Source: Sci Rep. 2016 Mar 7;6:22694. doi: 10.1038/srep22694 (PMC4780222; doi:10.1038/srep22694)
Supplement: Supplementary Information [file srep22694-s1.pdf]

A sandwich-type electrochemical immunosensor based on the biotin-streptavidin-biotin structure for detection of human immunoglobulin G

Yueyun Li[a,b], Yihe Zhang [a]*, Liping Jiang[b], Paul K. Chu[c], Yunhui Dong[b], Qin Wei[d]*

a. Beijing Key Laboratory of Materials Utilization of Nonmetallic Minerals and Solid Wastes, National Laboratory of Mineral Materials, School of Materials Science and Technology, China University of Geosciences, Beijing, 100083, P. R. China

b. School of Chemical Engineering, Shandong University of Technology, Zibo, 255049, P. R. China

c. Department of Physics & Materials Science, City University of Hong Kong, Tat Chee Avenue, Kowloon, Hong Kong, China

d. Key Laboratory of Chemical Sensing & Analysis in Universities of Shandong, School of Chemistry and Chemical Engineering, University of Jinan, Jinan, 250022, P.R. China

* Corresponding author:

Tel: +86-010-82323433; Fax: +86-010-82323433(Yihe Zhang)

Tel: + 86 531 82767872; Fax: + 86 531 82767367 (Qin Wei).

Table S1. Comparison between the proposed immunosensor and other sensors.

Methods	Linear ranges	Detection limits	References
Liquid Crystal and Silsesquioxane-Supported Gold Nanoparticles	0.1 ~ 0.9 ng/mL	0.076 ng/mL	[1]
Stretch−Stowage−Growth Strategy	0.001 ~ 50 ng/mL	0.4 pg/mL	[2]
Superparamagnetic Magnetite Nanoparticles	1.0 ~ 780 ng/m	0.28 ng/mL	[3]
multiplexed immunoassay	-	1.0 ng/mL	[4]
electrochemical detection	0.6 ~ 80 ng/mL	0.08 ng/mL	[5]
biosensor	1 ~ 10 ng/mL	-	[6]
High performance enzyme-linked immunosorbent assay	0.73~3000ng/L	-	[7]
biotin–streptavidin-amplified ed enzyme-linked immunosorbent assay	0.025~ 39.78 ng/ml	0.66 ng/mL	[8]
Polyclonal antibody-based enzyme-linked immunosorbent assay	0.73~3000.00 ng/mL	0.73 ng/mL	[9]
immunosensor	0.001 ~ 10 ng/mL	0.33 pg/mL	This work

Table S2. Analysis data sheet of the serum samples.

Initial HIgG concentration in sample (ng/mL)	Added HIgG concentration (ng/mL)	Measured concentration after addition (ng/mL)	Average (ng/mL)	RSD% (n=5)	Recovery rate (%, n=5)
0.95	1.00	1.94,1.98,2.01,1.93, 1.90	1.952	2.2	100.2
	5.00	5.87,5.69,6.08,5.93, 5.89	5.89	2.4	98.8
	10.00	10.75,11.12,10.85, 10.94,11.09	10.95	1.4	100.0

References

1 Zapp, E. *et al.* Troponin T Immunosensor Based on Liquid Crystal and Silsesquioxane-Supported Gold Nanoparticles. *Bioconjugate chemistry* **25**, 1638-1643 (2014).

2 Shao, K. *et al.* Stretch-stowage-growth strategy to fabricate tunable triply-amplified electrochemiluminescence immunosensor for ultrasensitive detection of pseudorabies virus antibody. *Analytical chemistry* **86**, 5749-5757, doi:10.1021/ac500175y (2014).

3 Huang, G., Deng, B., Xi, Q., Tao, C. & Ye, L. Surface Modification of Superparamagnetic Magnetite Nanoparticles and Its Application for Detection of Anti-CEA Using Electrochemiluminescent Immunosensor. *Med chem* **5**, 050-057 (2015).

4 Liu, D. *et al.* Multiplexed immunoassay biosensor for the detection of serum biomarkers — β-HCG and AFP of Down Syndrome based on photoluminescent water-soluble CdSe/ZnS quantum dots. *Sensors and Actuators B: Chemical* **186**, 235-243, (2013).

5 Feng, D. *et al.* Simultaneous electrochemical detection of multiple tumor markers using functionalized graphene nanocomposites as non-enzymatic labels. *Sensors and*

Actuators B: Chemical **201**, 360-368 (2014).
6 Wang, T., Yang, Z., Lei, C., Lei, J. & Zhou, Y. An integrated giant magnetoimpedance biosensor for detection of biomarker. *Biosensors and Bioelectronics* **58**, 338-344, (2014).
7 Yusakul, G. *et al.* High performance enzyme-linked immunosorbent assay for determination of miroestrol, a potent phytoestrogen from Pueraria candollei. *Analytica chimica acta* **785**, 104-110, doi:10.1016/j.aca.2013.04.053
8 (2013).
Bu, D., Zhuang, H., Zhou, X. & Yang, G. A heterogeneous biotin-streptavidin-amplified enzyme-linked immunosorbent assay for detecting tris(2,3-dibromopropyl) isocyanurate in natural samples. *Analytical*
9 *biochemistry* **462**, 51-59, doi:10.1016/j.ab.2014.06.003 (2014).
Yusakul, G. *et al.* Highly selective and sensitive determination of deoxymiroestrol using a polyclonal antibody-based enzyme-linked immunosorbent assay. *Talanta* **114**, 73-78, doi:10.1016/j.talanta.2013.04.011 (2013).
